# PLA-PEG Nanoparticles Improve the Anti-Inflammatory Effect of Rosiglitazone on Macrophages by Enhancing Drug Uptake Compared to Free Rosiglitazone

**DOI:** 10.3390/ma11101845

**Published:** 2018-09-27

**Authors:** Giovanna Giacalone, Nicolas Tsapis, Ludivine Mousnier, Hélène Chacun, Elias Fattal

**Affiliations:** Institut Galien Paris-Sud, CNRS, Univ. Paris-Sud, Université Paris-Saclay, 92296 Châtenay-Malabry, France; giovanna.giaccalone@u-psud.fr (G.G.); nicolas.tsapis@u-psud.fr (N.T.); ludivine.mousnier@u-psud.fr (L.M.); helene.chacun@u-psud.fr (H.C.)

**Keywords:** poly(lactide), nanoparticles, rosiglitazone, anti-inflammatory effect, macrophages

## Abstract

Among cardiovascular diseases, atherosclerosis remains the first cause of death in the United States of America and Europe, as it leads to myocardial infarction or stroke. The high prevalence of heart diseases is due to the difficulty in diagnosing atherosclerosis, since it can develop for decades before symptoms occur, and to the complexity of the treatment since targets are also important components of the host defenses. The antidiabetics thiazolidinediones, among which is rosiglitazone (RSG), have demonstrated anti-atherosclerotic effect in animal models, and are therefore promising candidates for the improvement of atherosclerosis management. Nevertheless, their administration is hindered by the insurgence of severe side effects. To overcome this limitation, rosiglitazone has been encapsulated into polymeric nanoparticles, which permit efficient delivery to its nuclear target, and selective delivery to the site of action, allowing the reduction of unwanted effects. In the present work, we describe nanoparticle formulation using polylactic acid (PLA) coupled to polyethylene glycol (PEG), their characterization, and their behavior on RAW264.7 macrophages, an important target in atherosclerosis treatment. RSG nanocarriers showed no toxicity on cells at all concentrations tested, an anti-inflammatory effect in a dose-dependent manner, up to 5 times more efficient than the free molecule, and an increased RSG uptake which is consistent with the effect shown. These biodegradable nanoparticles represent a valid tool to be further investigated for the treatment of atherosclerosis.

## 1. Introduction

Atherosclerosis is a chronic inflammatory disorder, the complications of which are the major cause of death among cardiovascular diseases [1]. The most severe aspect of atherosclerosis is the rupture of the plaques, which can result in myocardial infarction or stroke [2]. The formation of a plaque is due to the accumulation of lipids in a dysfunctional region of the arterial endothelium. Particularly, recruited monocytes that differentiate into macrophages and eventually give rise to foam cells play a key role in the development of inflammation [3].

Therefore, macrophage-targeting strategies represent a valid approach to selectively deliver anti-inflammatory drugs. Nanoparticles developed for medicine in the last decades could be an advantageous tool for two main reasons: at a cellular level, nanoparticles have been extensively demonstrated to be easily and efficiently taken up by circulating monocytes and macrophages without inducing any toxicity [4,5,6], thus improving the drug’s bioavailability. From a systemic point of view, nanocarrier distribution into the plaque target site is promoted by the enhanced permeability and retention effect [7], which is made possible in the case of atherosclerosis by the presence of dysfunctional endothelium. The use of this effect as a delivery strategy has already been extensively explored in nanomedical research for cancer therapy [8]. Recently, peroxisome proliferator-activated receptor γ (PPARγ), present on the nuclear surface of macrophage foam cells [9,10,11], has been identified as a down-regulator of macrophage activation and inflammation [12,13]. Thiazolidinediones are known PPARγ ligands which were marketed as insulin-sensitizers for the treatment of diabetes. Among them, rosiglitazone (RSG) has been recently shown to have an anti-atherosclerotic effect in mice [14]. Nevertheless, the use of this class of drugs is hindered by their severe adverse effects, such as congestive heart failure and myocardial ischemia [10,15]. Their delivery through a nanoparticulate system though would permit a targeted release of the cargo and therefore allow a reduction of the total administered dose, thus limiting the risk of side effects [16,17].

A few strategies have been recently proposed to encapsulate RSG into poly lactic-co-glycolic acid/polyvinyl alcohol (PLGA-PVA) nanoparticles [18], PLGA-polyethylene glycol (PEG) nanoparticles with a targeting peptide [19], poly(carbonate-co-lactide) nanoparticles [20], and cationic lipid emulsions [21]. In this work, we present the encapsulation of rosiglitazone into polymeric nanoparticles made of polylactic acid coupled to PEG (PLA-PEG). Although other polymers have been considered for macrophage targeting [22,23], PLA has been chosen because of its known biocompatibility [24] and PEG has been coupled to the polymer to provide stealth features to the resulting nanocarriers [25]. This long circulating effect and reduced liver uptake makes them accessible to other tissues, including inflammatory sites that are rich in macrophages in which uptake of PLA-PEG nanoparticles was demonstrated [26]. Nanoparticles have been characterized in terms of size, surface charge, and encapsulation efficiency. Their RSG release in physiological medium has been evaluated as well as their anti-inflammatory behavior and uptake by RAW264.7 macrophage cells.

## 2. Materials and Methods

### 2.1. Materials

Rosiglitazone (RSG), sodium cholate hydrate, poly(vinyl alcohol) (PVA), phosphate buffered saline (PBS), cell culture reagent MTT (3-(4,5-dimethylthiazol-2-yl)-2,5-diphenyltetrazolium bromide), and Lipopolysaccharides (LPS) were purchased from Sigma-Aldrich (Saint-Quentin-Fallavier, France). Radioactive rosiglitazone [benzylidene-3H] was obtained from ARC (Washington, DC, USA). DL-lactide was purchased from Biovalley, Polysciences Inc. (Warrington, PA, USA). Poly(ethylene glycol) methyl ether (OH-PEG-OCH_3_, average M_n_ = 5000 g/mol), stannous 2-ethyl hexanoate (stannous octoate, Sn(Oct)_2_), dried toluene, poly(vinyl alcohol) (PVA) (M_w_ = 30,000–70,000 g/mol, 87–90% hydrolyzed), D_2_O were provided by Sigma-Aldrich (Saint-Quentin-Fallavier, France). Deuterated chloroform (CDCl_3_) and deuterated acetone (acetone-d) were obtained from Euriso-top (Saint-Aubin, Essonne, France). All solvents were purchased from Carlo Erba (Val-de-Reuil, France). Water was purified using a RIOS/Synergy system from Millipore (Molsheim, France). NMR sample tubes and coaxial inserts were obtained from CortecNet (Bretonneux, France).

### 2.2. PLA-PEG Synthesis and Characterization

To a two-neck round-bottom flask previously flame-dried and under a flow of inert gas, DL-Lactide, HO-PEG-OCH_3_, and stannous octoate (1.5 mol PEG/mol stannous octoate) were dissolved into 10 mL of anhydrous toluene, and placed into an oil bath at 125 °C. Polymerization was carried out under stirring for 55 min. The reaction was stopped by simply dipping the flask in cold water. After evaporation of toluene under vacuum, the polymer was dissolved into chloroform and precipitated into cold diethylether. Then, the polymer was resuspended in tetrahydrofurane and precipitated into water before freeze drying for 24 h using an Alpha-1-2 LD apparatus (Christ, Coueron, France), leading to a white powder. The molecular weight was estimated by ^1^H NMR spectroscopy and the dispersity was evaluated by size exclusion chromatography (SEC). ^1^H NMR spectroscopy was performed in 5 mm diameter tubes in CDCl_3_ on an Avance-300 (300 MHz) spectrometer (Bruker, Billerica, MA, USA). SEC was performed at 30 °C with two columns from Polymer Laboratories (PL-gel MIXED-D; 300 × 7.5 mm) and a differential refractive index detector (Spectrasystem RI-150, Thermo Electron Corp., Waltham, MA, USA), using chloroform as an eluent, a Waters 515 pump at a flow rate of 1 mL/min, and toluene as a flow-rate marker. The calibration curve was based on poly(methyl methacrylate) (PMMA) standards from Polymer Laboratories. Lactide conversion ≥ 95% (^1^H NMR). ^1^H NMR (300 MHz, CDCl_3_, *δ*, ppm): 5.10–5.28 (CHCH_3_COO), 3.64 (OCH_2_CH_2_), 1.52–1.61 (CHCH_3_COO).

### 2.3. Nanoparticle Formulation and Characterization

Nanoparticles were formulated following two known protocols, namely emulsion-evaporation or nanoprecipitation. For the emulsion-evaporation method, 100 mg of PLA-PEG and 10 mg of rosiglitazone were dissolved in 4 mL acetone: dicholoromethane (1:1) or ethyl acetate:dichloromethane (1:1). The organic phase was then emulsified with 20 mL of 1.5% sodium cholate solution using a vortex for 1 min followed by sonication (Branson Sonifier, Danbury, CT, USA) for 1 min at 30% amplitude over ice. The solvent was evaporated by magnetic stirring at 300 rpm in a thermostated bath at 20 °C for 4 h.

For the nanoprecipitation method, 100 mg of PLA-PEG and different amounts of rosiglitazone (4, 7 and 10 mg) were dissolved in 10 mL of acetone or acetonitrile (ACN). Twenty mL of 1.5% sodium cholate solution were added with a syringe and needle (21G) using a syringe pump (11 Plus, Harvard Apparatus, Les Ulis, France), at 7 mL/min under vigorous stirring. The solvent was evaporated under reduced pressure in a bath at 30 °C using a rotary evaporator (Büchi, Flawil, Switzerland) cooled at −10 °C. All nanoparticles by nanoprecipitation have been prepared also on smaller scales, namely starting with 50 or 25 mg polymer.

For both techniques, nanoparticles were filtered through 0.45 µm PVDF filters and purified from free drug and polymer by ultracentrifugation at 27,400× *g* for 1 h at 4 °C. Pellets were resuspended in water during 10 min of vortex. Nanoparticle size and polydispersity index were determined in triplicate by dynamic light scattering using a NANO ZS (Malvern Instruments, Malvern, UK) at a 173° scattering angle. Prior to size measurements, nanoparticles were diluted 1:6 in water to obtain a suspension at 0.83 mg/mL polymer. Measurements were performed at 20 °C for 60 s. The surface charge of nanoparticles was evaluated by measuring the zeta potential on the same instrument by previously diluting the samples 1:3 in a 1 mM NaCl solution.

### 2.4. Encapsulation Efficiency

The amount of RSG encapsulated into the nanoparticles was determined through UV-Vis measurements on a LS25 spectrophotometer (Perkin Elmer, Villebon-sur-Yvette, France). Nanoparticles were separated from free molecules through ultracentrifugation (50,400× *g* for 1 h at 4 °C) and then dissolved by diluting them 1:10 in acetonitrile. A calibration curve of RSG in the same solvent ratio was previously performed, by measuring the intensity of the peak at λ = 246 nm. Encapsulation efficiency (EE) was then obtained as the percentage of the difference between the current concentration and the one at the beginning of the preparation. Drug loading (DL) corresponds to the weight of RSG with respect to the total weight of the nanoparticle (polymer+RSG) expressed as a percentage.

### 2.5. Release Studies

Radioactive nanoparticles with a drug loading of 2.1% (*w*/*w*) were prepared by nanoprecipitation by adding 12 µCi of radiolabelled rosiglitazone to the organic solution before nanoparticle formation. After purification, nanoparticles were diluted to yield a rosiglitazone concentration 10 times lower than its solubility in the release medium (PBS) (1.5 µg/mL, sink conditions). Nanoparticle aliquots were incubated under agitation at 37 °C and withdrawn after scheduled times. Nanoparticles and release medium were separated through ultracentrifugation (50,400× *g* for 1 h at 4 °C) and their radioactivity content was determined separately after addition of the liquid scintillation cocktail Ultima Gold (Perkin-Elmer, Villebon-sur-Yvette, France), using a Beckman Coulter (LS 6500 Multi-Purpose Scintillation Counter, Villepinte, France) instrument.

### 2.6. In Vitro Studies on Cells: Toxicity, Internalization, and Anti-Inflammatory Potential

RAW264.7 mouse macrophages were obtained from ATCC (Manassas, VA, USA). They were cultured in DMEM medium (Sigma-Aldrich, Saint-Quentin-Fallavier, France), supplemented with 10% fetal bovine serum (Lonza, Basel, Switzerland), penicillin (100 IU/mL), and streptomycin (100 µg/mL) (Lonza, Basel, Switzerland). Cells were maintained in a humidified incubator with 95% air/5% CO_2_ at 37 °C. Cells were split twice a week at a ratio between 1/6 and 1/8 after detachment by scraping. After thawing, cells were used for experiments from passage 6 to 15.

Nanoparticles toxicity towards RAW264.7 cells was determined by measuring mitochondrial activity using the 3-[4,5-dimethylthiazol-2-yl]-3,5-diphenyltetrazolium bromide (MTT) assay [27]. Cells were seeded in a 96-well plate at a density of 8000 cells/well and incubated for 24 h. Nanoparticles at concentrations ranging from 0.8 µg/mL to 2.4 mg/mL were added on the cells for 24 h and subsequently replaced by a 0.5 mg/mL MTT solution for 2 h. MTT was removed and DMSO was added to permeabilize the cells and dissolve the crystals. Absorbances were measured using a Labsystems Multiskan MS plate reader at 570 nm.

Inflammation of RAW264.7 cells was evaluated by the detection of 4 cytokines (MCP-1, TNF-α, IL-10, IL-6) using the BD Cytometric Bead Array (CBA) Mouse Inflammation Kit (BD). Cells were pre-incubated for 24 h and then exposed for 3 h to lipopolysaccharides (LPS) at 0.1 µg/mL to induce inflammation. Nanoparticles or free RSG at different concentrations were then added to the cells and incubated for 24 h, after which the supernatants were collected for cytokines measurement. The samples were prepared following manufacturer instructions and analyzed using a BD Accuri C6 flow cytometer (Becton Dickinson, Rungis, France). Statistical analysis was performed using a unilateral Student’s *t*-test as provided by Microsoft Excel.

Nanoparticle uptake by macrophages was evaluated by using radiolabeled RSG. Free RSG and nanoparticles at 3 different concentrations (7.6, 59, and 227 µM of RSG) were added for 24 h to the RAW264.7 cells after 3 h incubation with LPS. Subsequently, supernatants and cells were separated, cells were rinsed with PBS and their radioactivity content was measured separately.

## 3. Results and Discussion

The aim of the present study was to optimize the encapsulation of RSG into PLA-PEG nanoparticles and to evaluate their capacity to improve drug cellular uptake and anti-inflammatory activity of RAW264.7 macrophages.

### 3.1. Nanoparticle Formulation

Nanoparticles were first obtained using the emulsion-evaporation technique. To achieve the simultaneous solubilization of the polymer and RSG, 1:1 mixtures of dichloromethane (DCM):acetone or DCM:ethyl acetate were used. Nanoparticle diameter was measured immediately after centrifugation to separate them from free drug. Nanoparticles obtained after purification display a diameter around 125 nm, a polydispersity index (PDI) of 0.15 and are negatively charged (−18 mV) independently of the solvent mixture used (Table 1).

Nanoparticles were also obtained by the nanoprecipitation technique using either acetonitrile (ACN) or acetone to solubilize the polymer–drug mixture. Consistently with literature [28], smaller nanoparticles were obtained with this method: around 115 nm when using ACN (Table 2) and surprisingly smaller (around 80 nm) when they were prepared with acetone (Table 3). It is generally known that the use of more polar solvents like acetone results in smaller nanoparticles, due to the easy diffusion of the solvent in the aqueous phase. Yet, ACN has a higher polarity index than acetone [29] and should therefore diffuse more rapidly in the water phase. Nevertheless, other parameters affect the final nanoparticle size, the affinity between the solvent and the polymer, for instance [9]. This is the reason why in some cases like the present one smaller nanoparticles can be obtained with acetone [9,30].

For further encapsulation efficiency studies, different starting amounts of RSG were tested (4, 7, and 10 mg) without significantly affecting nanoparticles characteristics (Table 2 and Table 3). For both solvents, less debris or aggregates were observed with nanoprecipitation as compared to the solvent evaporation technique.

### 3.2. Encapsulation Efficiency

**Figure 1:**
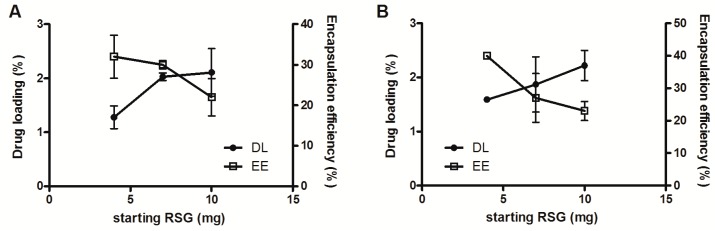
RSG drug loading (DL, left axis) and encapsulation efficiency (EE, right axis) in nanoparticles prepared with acetonitrile (**A**) or acetone (**B**) by nanoprecipitation as a function of RSG starting concentration.

Encapsulation efficiency was determined for all formulations. Nanoparticles were separated from free drug by ultracentrifugation and dissolved in acetonitrile to assay the entrapped RSG. Results are expressed as encapsulation efficiency and drug loading (Figure 1 and Table 4). Drug loading is calculated as the ratio between the actual amount of RSG entrapped and the sum of the initial amount of polymer plus the actual amount of RSG entrapped. Actual values might be higher as possibly not the totality of the polymer is finally involved in the nanoparticle formation, especially in the case of emulsion-evaporation, for which a lot of debris was removed by filtration as evidenced by the difference in size before and after filtration (Table 1).

As shown in Figure 1, the encapsulation of RSG in nanoparticles obtained by nanoprecipitation using ACN (A) or acetone (B) as solvent present a similar trend, reaching a drug loading of around 2% in both cases. For ACN nanoparticles, the drug loading seems to level off at 2% already with a starting amount RSG of 7 mg, which corresponds to encapsulation efficiencies of 20–30%. In the case of acetone, the drug loading seems to increase with increasing starting amount, reaching slightly levels above 2% with a starting amount of RSG of 10 mg. These relatively low drug loadings achieved are nevertheless in agreement with literature as they strongly depend on drug–polymer interactions [9,20,23,30].

As it can be clearly seen from Table 4, nanoprecipitation leads to higher encapsulation efficiency and drug loading compared to emulsion-evaporation. Therefore, nanoparticles obtained by nanoprecipitation were selected for further experiments. The two solvents were very similar in terms of EE and DL, so the acetone formulation was chosen for safety reasons, being a class 3 solvent whereas acetonitrile is a class 2 (International Council for Harmonisation (ICH) guideline Q3C (R6) on impurities: guideline for residual solvents EMA/CHMP/ICH/82260/2006, 6 December 2016). Ten mg RSG starting amount was used in the following experiments since it results in the highest DL.

### 3.3. RSG Release from Nanoparticles

**Figure 2:**
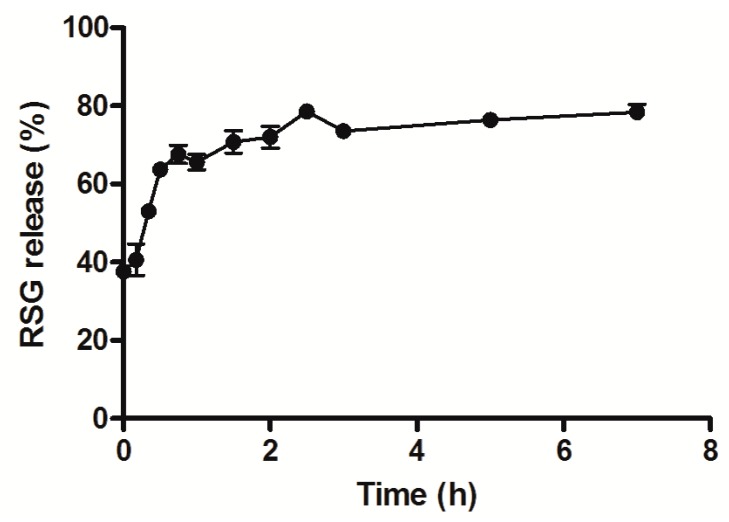
RSG release from nanoparticles in PBS at 37 °C, under sink conditions.

Release experiments were then performed to evaluate the behavior of the drug and assess the extent of the burst effect. In other words, it was important to analyze how much of the drug would be released before the nanoparticle reached the target. PBS was chosen as typical release medium that mimics physiological conditions. Indeed, nanoparticles presented a burst release effect of almost 70% during the first hour of incubation (Figure 2), most likely due to surface-associated molecules [31]. This first part of the curve corresponds to a Fickian diffusion as suggested by the calculated release exponent value of the Korsmeyer–Peppas equation (n = 0.41; 0.43 corresponds to Fickian diffusion from spheres). Subsequently, the release is slower, reaching a sort of plateau around 80% for the following hours, possibly due to the diffusion of the drug encapsulated in the inner core of the nanoparticles. This release behavior is commonly observed and in agreement with literature about polyester nanoparticles [10].

### 3.4. Cell Viability in the Presence of RSG Nanoparticles

**Figure 3:**
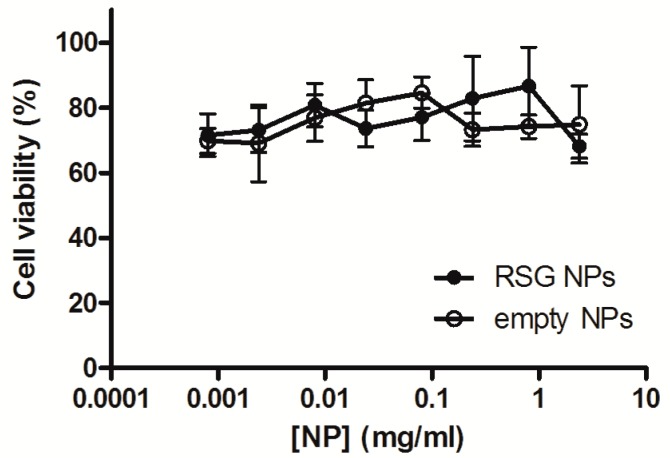
RAW264.7 cells’ viability after 24 h incubation with RSG NPs and blank NPs at different concentrations.

In the context of the atherosclerotic disease, plaque macrophages represent a relevant target of anti-inflammatory drugs as they play a key role in many processes involved in the progression of the disease. Among others, they can undergo apoptosis and therefore contribute to the plaque necrotic core, are involved in the activation of immune responses, promote the platelet aggregation on the inflammation site, and produce metalloproteinases which are responsible for the thinning of the fibrous cap. Therefore, RAW264.7 murine macrophages have been chosen to test nanoparticles efficiency in reducing the inflammation. Preliminarily, it is pivotal to assess the nanoparticles’ safety towards the chosen cell line. An MTT test was performed which evaluated the mitochondrial activity of treated cells. Cells were incubated 24 h with RSG nanoparticles and the corresponding empty nanoparticles at different concentrations. As depicted in Figure 3, no significant difference in cell viability was found among all concentrations tested. Both formulations grant a cell viability of around 70% for concentrations between 8 µg/mL to 2.4 mg/mL. These nanoparticles can be considered safe towards RAW264.7 cells even at relatively high concentrations and have therefore been tested further for their anti-inflammatory activity.

### 3.5. Inflammation Evaluation in RAW264.7 Cells

**Figure 4:**
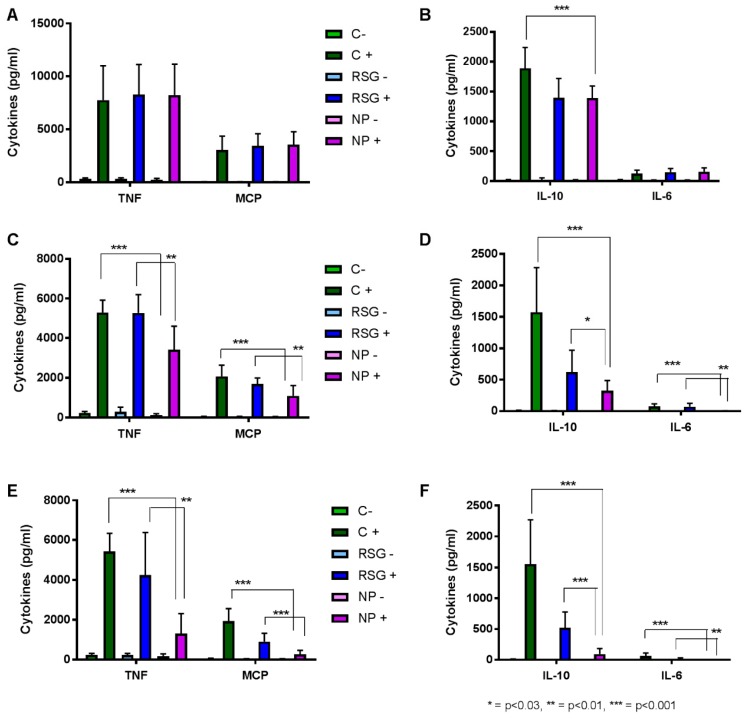
TNF, MCP (**A**,**C**,**E**), IL-10, and IL-6 (**B**,**D**,**F**) concentrations in supernatants of RAW264.7 cells after 24 h exposure to cell medium (C-), 0.1 µg/mL LPS (C+), free RSG (RSG-), free RSG and LPS (RSG+), NPs (NP-), NPs and LPS (NP+). Rosiglitazone and corresponding NP concentrations are 7.6 µM (**A**,**B**), 59 µM (**C**,**D**), 227 µM (**E**,**F**).

After having established the non-toxicity of PLA-PEG RSG nanoparticles towards RAW264.7 cells, their anti-inflammatory effect has been investigated and compared to that of the free molecule. Inflammation in cells was quantified as the amount of cytokines released in their supernatant, namely tumor necrosis factor (TNF-α), monocyte chemoattractant protein-1 (MCP-1), interleukin-6 (IL-6), and interleukin-10 (IL-10). Cells were incubated for 24 h with different concentrations of RSG and corresponding nanoparticles. Inflammation was previously developed by a 3 h incubation with 0.1 µg/mL LPS. As can be seen by the cytokines production (Figure 4), this protocol was adequate to produce inflammation in cells. At low RSG concentration (Figure 4A,B), almost no difference can be observed among all the LPS-treated cells, whether they further received the addition of RSG or NPs or not. Only exception is IL-10, where NPs were able to reduce the production of this cytokine. As RSG concentration is increased (Figure 4C,D), NPs effect can be seen as compared to non-treated cells (LPS+) as well as to free RSG-treated cells (RSG+). This effect is even more obvious and significant at the highest RSG concentration tested (Figure 4E,F). In the case of IL-10, NPs can reduce its expression by 5 and 16 times more than the free RSG and the medium, respectively (Figure 4F). Therefore, PLA-PEG RSG nanoparticles show potential in efficiently reducing cytokines levels in inflamed macrophages and can be considered for further in vivo evaluations.

### 3.6. Free RSG and NP Uptake by RAW264.7 Cells

**Figure 5:**
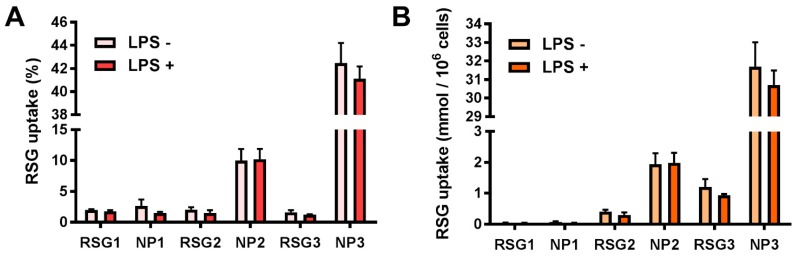
RSG uptake by RAW264.7 cells after 24 h incubation with free RSG or NPs at 3 different concentrations: RSG1, NP1 = 7.6 µM; RSG2, NP2 = 59 µM; RSG3, NP3 = 227 µM. Uptake expressed as (**A**) percentage of RSG added amount and (**B**) mmol per million of cells.

To explain the mechanism of the improved nanoparticles’ efficiency in reducing the inflammation as compared to the free molecule, an uptake study has been performed for the evaluation of intracellular levels of RSG in both cases. Radioactive RSG has been used as a tracer and the same protocol as for the cytokines detection has been applied. As Figure 5 shows, no internalization difference between free RSG and NPs can be seen for the smallest RSG concentration. However, the difference in the internalization increases as the RSG concentration does, up to 40% NP RSG uptake as compared to 2% for the free molecule, when the highest concentration was tested. This corresponds to 30 mmol of RSG delivered per million of cells (Figure 5B). The increased RSG uptake in the case of NPs correlates with their superior efficiency in reducing the inflammation, due to the greater amount of molecule delivered inside the cells at its site of action. Furthermore, the presence or absence of LPS does not affect the cell’s capability to take up nanoparticles.

## 4. Conclusions

In this paper, we presented the formulation of rosiglitazone-loaded PLA-PEG nanoparticles prepared with different techniques and conditions. All nanoparticles have been characterized in terms of size, surface charge, and encapsulation efficiency. Nanoparticles prepared by nanoprecipitation in the presence of acetone were selected as the best formulation for further investigation. These nanoparticles showed no toxicity on RAW264.7 cells even at high concentrations as proved by the MTT test and were able to reduce cytokine production by 16 times at a lower level than the control. Finally, the enhanced anti-inflammatory effect could be explained by increased drug uptake into the cells when rosiglitazone is encapsulated into nanoparticles, as compared to the free molecule. Therefore, these delivery systems showed their potential as carriers for anti-inflammatory drugs, which could be further developed and thus lead to an improvement in the current atherosclerosis treatment.

## Figures and Tables

**Table 1:** Size, PDI, and zeta potential of nanoparticles prepared by emulsion-evaporation technique (10 mg RSG starting amount).

Solvent Mixture	Size (nm) ± SD	Polydispersity Index ± SD	Zeta Potential (mV)
DCM:acetone 1:1	123 ± 7	0.15 ± 0.06	−17 ± 6
DCM:ethyl acetate 1:1	130 ± 5	0.16 ± 0.05	−18 ± 7

**Table 2:** Size, PDI, and zeta potential of nanoparticles prepared by nanoprecipitation technique using acetonitrile.

Drug Amount	Size (nm) ± SD	Polydispersity Index ± SD	Zeta Potential (mV)
RSG 4 mg	115 ± 2	0.06 ± 0.01	−20 ± 1
RSG 7 mg	111 ± 3	0.09 ± 0.02	−21 ± 1
RSG 10 mg	115 ± 4	0.12 ± 0.02	−15 ± 4

**Table 3:** Size, PDI, and zeta potential of nanoparticles prepared by nanoprecipitation technique using acetone.

Drug Amount	Size (nm) ± SD	Polydispersity Index ± SD	Zeta Potential (mV)
RSG 4 mg	79 ± 1	0.11 ± 0.01	−23 ± 3
RSG 7 mg	81 ± 1	0.10 ± 0.01	−17 ± 6
RSG 10 mg	82 ± 4	0.14 ± 0.02	−28 ± 1

**Table 4:** RSG encapsulation efficiency (EE) and drug loading (DL) in different NP formulations prepared by emulsion-evaporation or nanoprecipitation, with different RSG starting amounts (4, 7, or 10 mg). DCM = dichloromethane, ACN = acetonitrile.

Solvent Mixture	Emulsion-Evaporation		Nanoprecipitation	
	EE (%)	DL (%)	EE (%)	DL (%)
Acetone/DCM 10 mg	4.7	0.47		
ethyl acetate/DCM 10 mg	5.7	0.56		
ACN 10 mg			22	2.1
ACN 7 mg			30	2.0
ACN 4 mg			32	1.3
Acetone 10 mg			22	2.1
Acetone 7 mg			23	1.6
Acetone 4 mg			41	1.6
